# A Tale of Three Dieulafoy Lesions: A Case Report and Review of the Literature

**DOI:** 10.7759/cureus.8365

**Published:** 2020-05-30

**Authors:** Shivantha Amarnath, Subash Ghimire, Hafiz M Khan

**Affiliations:** 1 Internal Medicine, Staten Island University Hospital, Northwell Health, Staten Island, USA; 2 Internal Medicine, Guthrie Clinic, Robert Packer Hospital, Sayre, USA; 3 Gastroenterology and Hepatology, Guthrie Clinic, Robert Packer Hospital, Sayre, USA

**Keywords:** dieulafoy lesion, upper gastrointestinal bleed, hematemesis, liver cirrhosis

## Abstract

A Dieulafoy lesion (DL) is a rare cause of non-variceal upper gastrointestinal hemorrhage. It is a large submucosal artery that lies in close proximity to the mucosal surface without any evidence overlying superficial ulcers. DLs can be found anywhere within the gastrointestinal (GI) tract, but are frequently encountered within the stomach. Most cases documented in the literature only describe isolated, single DLs occurring within the GI tract. Herein, we describe an unusual case of an elderly female with a medical history of compensated alcoholic liver cirrhosis who presented with massive hematemesis and endoscopy unveiled three DLs within the gastric cardia as the source of hemorrhage. The bleeding was successfully managed using novel endoscopic modalities such as Hemospray (Cook Medical, Bloomington, IN). We also provide an updated literature review on the diagnosis, pathophysiology as well as recent advances in the management of DLs.

## Introduction

A Dieulafoy lesion (DL) is a large, caliber-persistent submucosal artery with a diameter of 1-3 mm. It is located in close proximity to the mucosal surface and is subject to injury and consequent bleeding [1-3]. The literature reveals that isolated DLs most commonly occur either in the bronchial tree or the gastrointestinal (GI) tract [1]. The latter most commonly involves the stomach (75%-95% of cases), especially within 6 cm of the gastroesophageal junction [1]. Arterial supply of this region comes directly from the branches of the left gastric artery [4]. There are also isolated cases of DLs that occur in the duodenum, colon, and even the rectum [5]. Bleeding from DLs mostly occurs in males than females (2:1), especially in the fifth decade of life [5,6].

DLs account for 6% of all causes of upper GI hemorrhage [1]. Patients mostly present with acute, massive, intermittent painless melenic stools and hematemesis [5,7]. All recent studies reported in the literature have only demonstrated isolated, single DLs uncovered in a patient whilst the presence of multiple DLs in a single patient is extremely rare and the literature only provides very few case reports in the pediatric population. For instance, a case report described a 15-year-old child who presented with melena and hematemesis, and endoscopy revealed two synchronous DLs in the stomach and jejunum [8]. To date, our case is the first to be reported in the literature where an elderly female with compensated alcoholic cirrhosis presented with multiple episodes of hematemesis and acute blood loss anemia and immediate endoscopy revealed three DLs in the gastric cardia.

## Case presentation

A 73-year-old female with a past medical history of compensated alcoholic liver cirrhosis with small nonbleeding esophageal varices on screening endoscopy (EGD) carried out three months ago, hypertension and coronary artery disease with the placement of a drug-eluting stent in the left anterior descending artery (LAD) three weeks ago, on dual antiplatelet therapy presented to the emergency room with multiple episodes of hematemesis and melena. On admission, the patient had orthostatic vital signs, elevated troponins peaking at 1.2 ng/mL likely from demand ischemia, and acute blood loss anemia requiring blood transfusions due to drop in hemoglobin from baseline of 12.5 g/dL to 7.8 g/dL. Platelet count was reactively elevated to 259 K/uL from a baseline of 140 K/uL, and international normalized ratio (INR) was 1.6. Given the severity of bleeding and high risk for cardiac events due to coronary artery disease and recently placed LAD stent, she was admitted to the intensive care unit and urgent EGD was performed within six hours of admission, after initial resuscitation and stabilization of hemodynamics.

**Figure 1:**
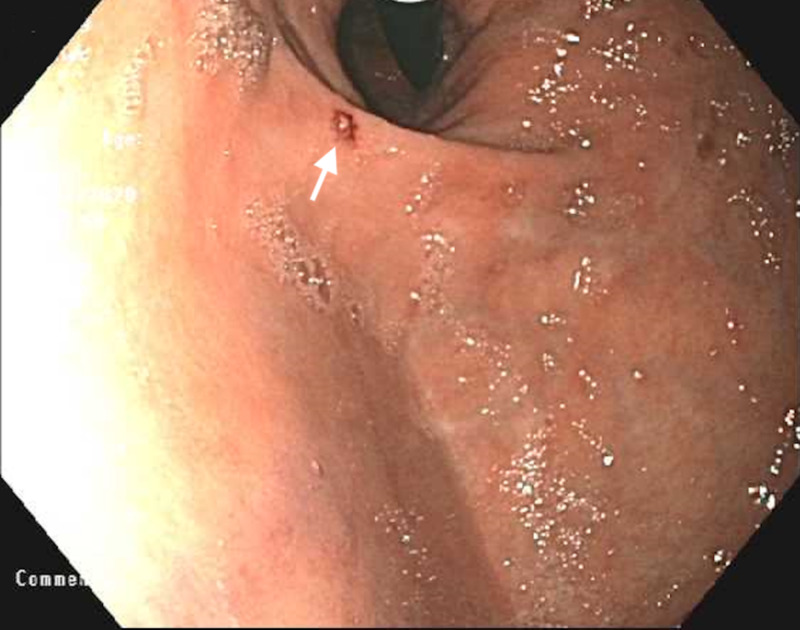
Endoscopic retroflexed view of a Dieulafoy lesion (arrow) and hiatal hernia

**Figure 2:**
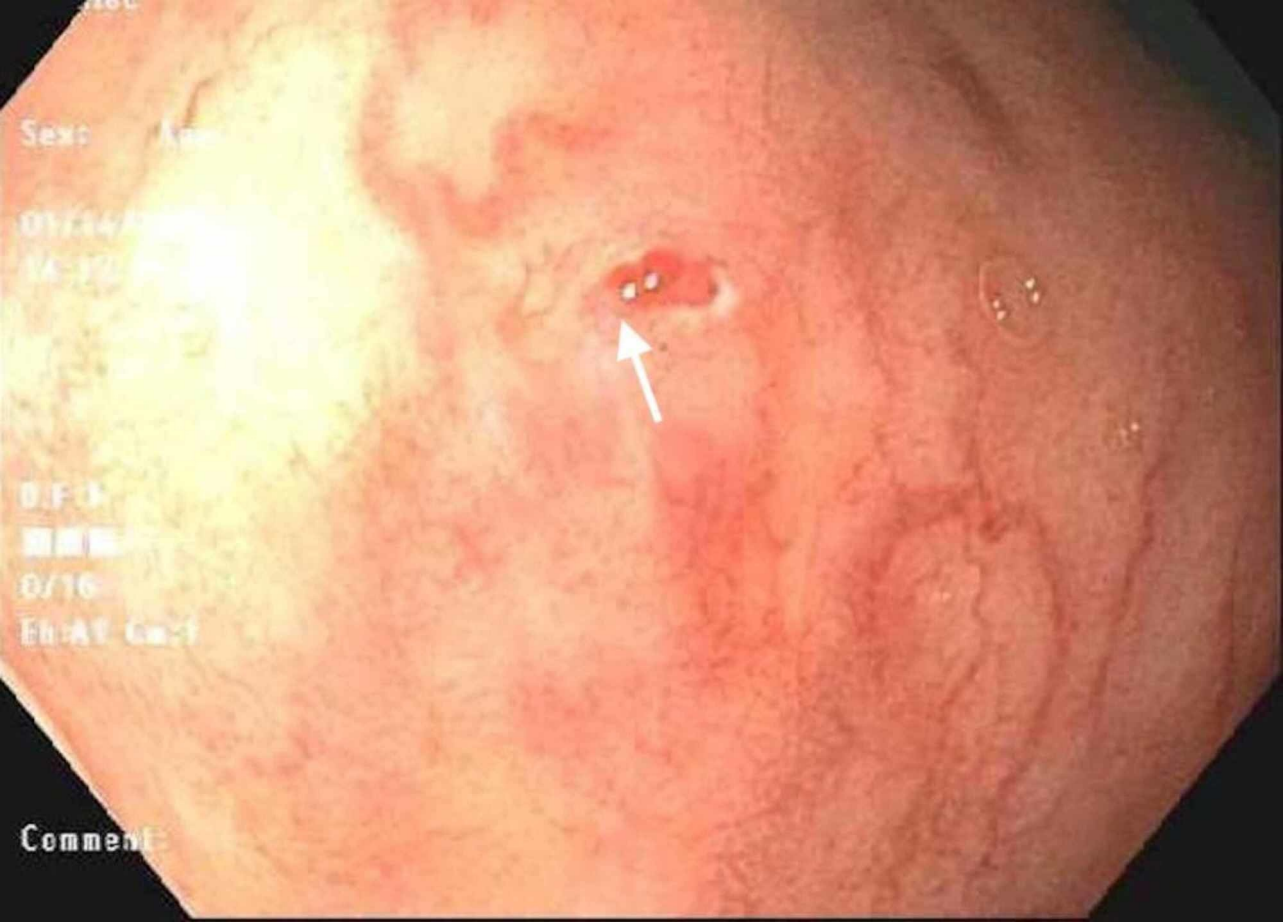
Endoscopic view of the second Dieulafoy lesion (arrow)

**Figure 3:**
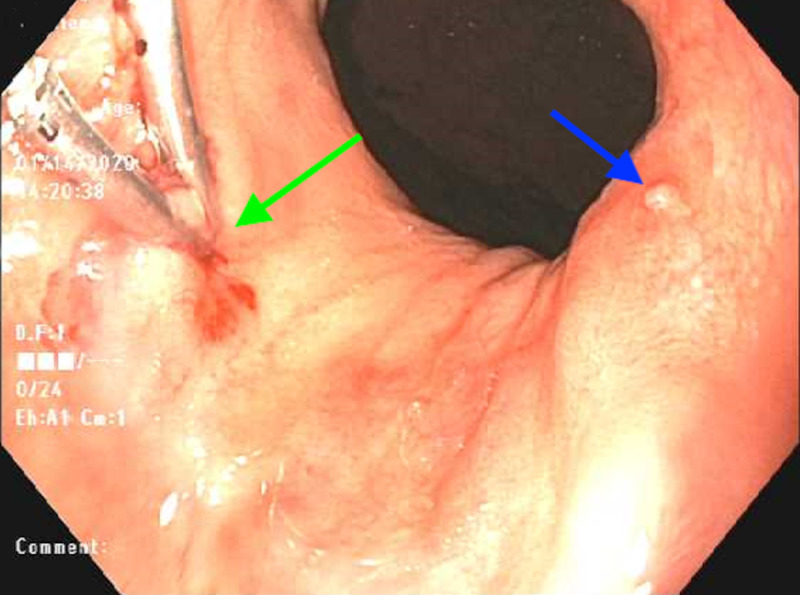
Endoscopic view of a Dieulafoy lesion to which two hemostatic clips have been applied (green arrow). Evidence of a third Dieulafoy lesion (blue arrow)

**Figure 4:**
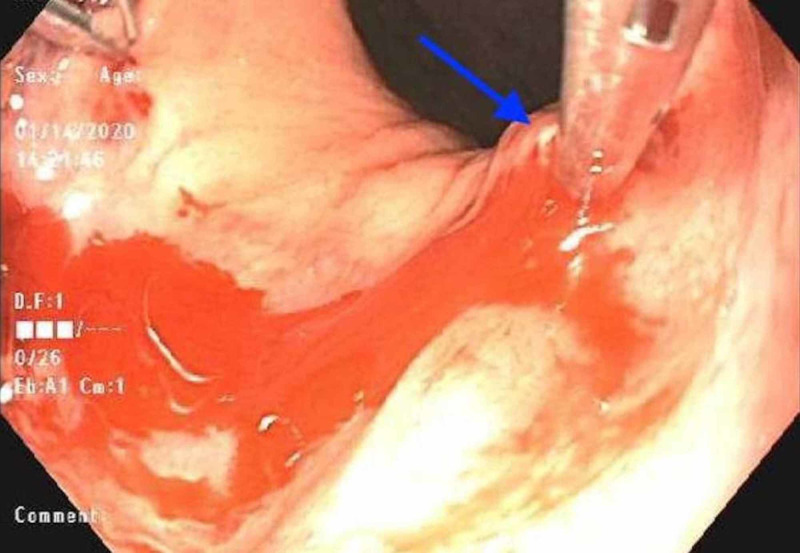
Endoscopic view of a single hemostatic clip applied to third Dieulafoy lesion with evidence of persistent bleeding (blue arrow)

**Figure 5:**
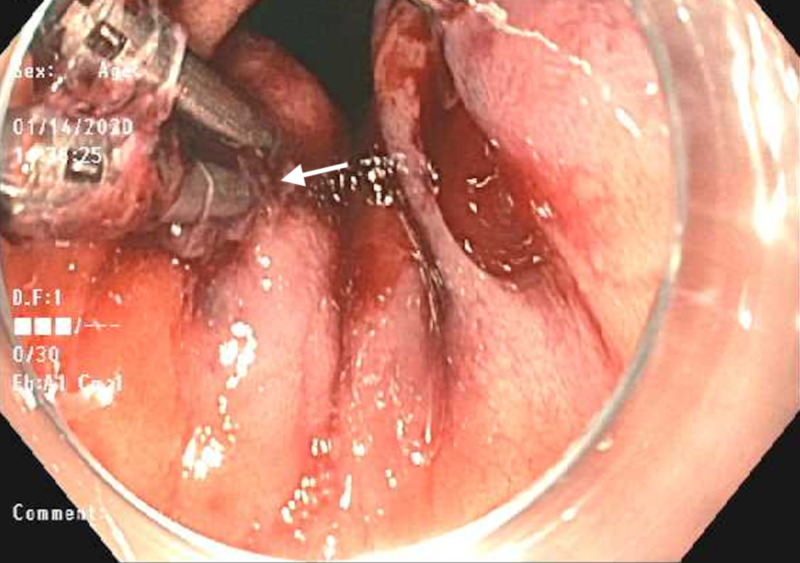
Endoscopic view of hemoclips applied to two Dieulafoy lesions with evidence of persistent oozing

**Figure 6:**
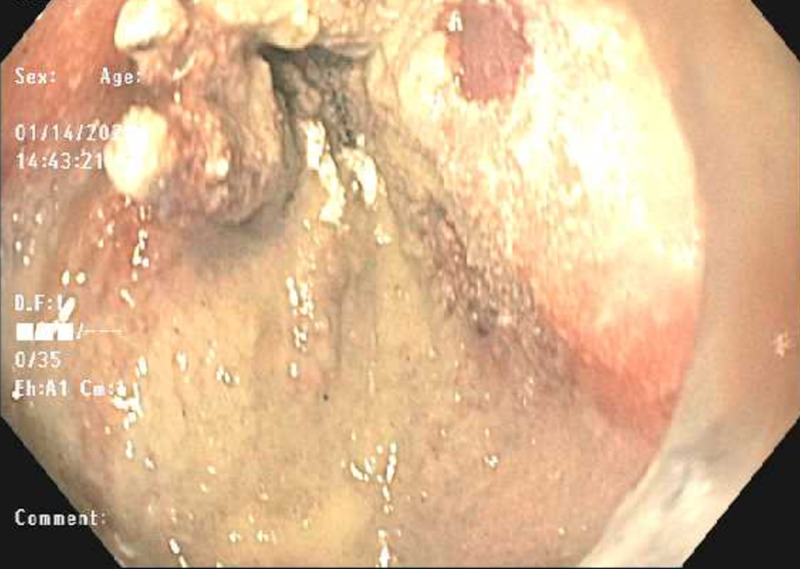
Endoscopic view of the site of Dieulafoy lesions after application of Hemospray and successful hemostasis

EGD revealed a large blood clot in the gastric fundus that was removed using a Roth Net (Steris, Mentor, OH) and careful examination of the upper GI tract unveiled three DLs in the gastric cardia (Figures 1-3). The use of electrocautery was considered a high risk of further bleeding since the patient was on dual antiplatelet therapy. Partial hemostasis was achieved using epinephrine injection around the lesions and endoclips were placed on each lesion with blood still oozing underneath all three lesions (Figures 4, 5). This was thought to be due to the antiplatelet effects of aspirin and clopidogrel. Hemospray (TC-325) (Cook Medical, Bloomington, IN) was then used as a rescue therapy resulting in complete hemostasis (Figure 6). The patient was restarted on dual antiplatelet therapy within 24 hours after consultation with cardiology due to an increased risk of stent thrombosis. The patient remained hospitalized for 72 hours without evidence of rebleeding. She was subsequently discharged from the hospital. She was seen in the outpatient clinic at two weeks and four weeks after discharge and her hemoglobin normalized to 12.1 g/dL and did not endorse any further episodes of bleeding. 

## Discussion

DLs are an uncommon cause of non-variceal upper GI bleeding. Two hypotheses to explain the reason for spontaneous bleeding from these lesions have been proposed: The pulsatile nature of the caliber-persistent submucosal artery can cause the overlying mucosa to erode and the vessel to protrude through the mucosa and bleed as a direct consequence of arterial wall injury from the gastric contents [2]. An alternate theory is that age-related gastric wear and tear promotes thrombosis within the submucosal artery, which eventually leads to necrosis of the affected vessel [7].

The risk of bleeding is highest in patients with comorbid conditions like peptic ulcer disease, cardiovascular disease, chronic kidney disease, hypertension, type 2 diabetes mellitus, and chronic nonsteroidal anti-inflammatory drugs (NSAIDs), anticoagulants or antiplatelet use [6]. It is theorized that chronic erosive gastritis due to NSAIDs and antiplatelet use and subsequent necrosis of the arterial wall may lead to rupture of the submucosal vessel [5]. Shin et al. (2014) performed a case-control study that focused on identifying potential risk factors for bleeding DLs in 42 patients [9]. The results revealed that patients on antiplatelet agents are at higher risk for bleeding compared to NSAIDs. They also found that chronic alcohol use was also a risk factor for bleeding DLs. The incidence of initial bleed and recurrent bleeding is increased in patients with alcoholic liver disease and carries a high mortality rate [10]. One study proposed that chronic alcohol use damages the gastric mucosal layer and stimulates gastric acid secretion which in turn weakens the arteriolar wall [11]. This consequently results in vessel rupture and bleeding. The propensity of DLs to bleed is further increased by portal hypertension and coagulopathy among those with cirrhosis. 

Hence, our case presents a remarkable phenomenon where three synchronous DLs were identified in an elderly female patient with alcoholic cirrhosis. Her risk of bleeding may have been significantly increased by her alcohol use, chronic liver disease with coagulopathy, and the use of dual antiplatelet therapy. It is imperative that endoscopists should be aware of the fact that multiple DLs can be encountered when attempting to identify a source of bleeding in patients with liver cirrhosis.

Diagnosis is mostly made via direct endoscopic visualization of the DL and this procedure itself carries a 70% success rate at identifying the lesion. A pulsating vessel may become visible with surface mucosal erosion of about 1-5 mm and without any evidence of inflammation at the edge of the mucosal defect [5]. The endoscopic diagnostic criteria for DLs include the following [4]: active arterial spurting or micro-pulsatile streaming from a minute mucosal defect (< 3 mm); direct visualization of the protruding vessel with or without active bleeding within the mucosal defect with normal mucosa along the periphery; evidence of a densely adherent clot with a narrow attachment point to a mucosal defect or normal-appearing mucosa. However, there is a diagnostic dilemma where on occasions the lesion may be too small to be identified and this is further affected by the relatively normal appearance of the surrounding mucosa [2]. Additionally, endoscopy may fail to reveal the bleeding site due to excessive blood in the stomach [5]. Hence, it is not uncommon that multiple endoscopies are required before a diagnosis can be made. Tattooing the affected site during initial endoscopy may aid in the identification of the site, should bleeding recur [7].

Biopsies of the affected vessel are not often performed due to bleeding risk, but previous studies have shown that the affected artery appears normal on histology with occasional amyloid deposits [2]. There is often no evidence of ulceration or deep penetration into the muscularis propria [12]. The key feature that distinguishes DLs from gastric ulcers is the lack of sub-intimal fibrosis and mucosal inflammation [9].

Endoscopic intervention carries a 70% to 98% success rate of achieving hemostasis [4]. Endoscopic treatment options include the following: injection therapy using epinephrine; mechanical therapy involving hemoclips or banding; and thermal therapy using Argon Plasma Coagulation, monopolar or bipolar electrocoagulation [13]. Mechanical hemostasis is the safest and most effective approach [10]. Injection therapy alone is less effective as initial therapy, but in cases of massive bleeding, an epinephrine injection can be easily applied to the bleeding site to control the bleed and this can then be combined with mechanical or ablative therapy for successful hemostasis [10]. Endoscopic hemoclipping is more efficacious than injection therapy alone, especially in the proximal stomach with rebleeding rates of 0% vs 45.5% (5). Endoscopic band ligation is an effective option, especially when intervention with endoscopic hemoclipping has failed [12]. A meta-analysis performed by Barakat et al. (2018), included five studies with a total of 162 patients demonstrated that endoscopic band ligation and endoscopic hemoclip placement are both equally effective procedures for the management of DLs (OR: 1, 95% CI [0.96-1.05)] and have similar rates of hemostasis and rebleeding (OR: 0.37, 95% CI [0.12-1.09]) [3]. The primary benefits of implementing EBL include the following [12]: ease of application on DLs in the GE junction and posterior wall of the body of the stomach where the bands disrupt blood supply from the ligated bleeding vessel; significantly reduced perforation risk compared to other modalities. However, EBL is not without its limitations. For instance, there still remains a risk of ulcer formation on the surface of the ligated mucosa. Also, additional time is required to prepare the overtube or multi-ligating device [12]. Modern endoscopic interventions have remarkably reduced the mortality rate of DLs from 80% to 8.6%. Recently, several studies have documented the prominent role of Hemospray (TC-325) in the management of non-variceal upper GI bleeding. It is a powder that can be sprayed onto an active bleeding site and is able to tamponade the bleed up to 48-72 hours. It also acts by increasing clotting factor concentration with downstream activation of the coagulation cascade to initiate immediate clotting. The location and size of the bleeding site is a challenge often faced by many endoscopists and in these situations, utilization of the Hemospray has a significant advantage due to its ease of use. However, it only functions as an adjunctive treatment for other definitive traditional endoscopic modalities. A recent meta-analysis comprising of 20 studies incorporating roughly 1,280 patients with non-variceal upper GI hemorrhage demonstrated that the use of Hemospray carried a technical and clinical success of 97% (95% CI 94-98%, I2 = 47.72%) and 91% (95% CI 88-94%, I2 = 47.7%), respectively, compared to traditional hemostatic measures [14]. The risk of rebleeding (27%), and treatment failure (31%) were also low. It should also be noted that our patient was restarted on dual antiplatelet therapy due to his recent cardiac stent and did not have any further bleeding after achieving initial hemostasis. This case also demonstrates that the use of Hemospray is effective as adjunctive therapy in the management of bleeding from multiple DLs. It can be safely applied to the site of bleeding with minimal difficulty and should be considered as a tool for achieving complete hemostasis prior to pursuing other invasive strategies like surgery.

Despite the effective endoscopic intervention of DLs, there is still a 10% chance of rebleeding especially within the first 30 days of therapy [6]. The risk of rebleeding is higher when epinephrine injection is used alone, without added mechanical therapy or electrocautery [12]. If endoscopy fails to reveal an obvious etiology or control of the bleeding, then Angiography or Red Cell Scan may be performed. Angiography is often the best next step, should initial endoscopy fail to achieve hemostasis. This holds true especially in the colon where colonoscopic visualization is often affected by poor bowel preparation or excessive bleeding but also considered a good alternative if the bleeding was in the gastric cardia due to easy access to the left gastric artery. DLs often present as contrast extravasation from a normally appearing blood vessel from the submucosa [7]. Angiography is also the procedure of choice when the bleeding site is beyond the reach of the therapeutic endoscope and for patients who are not surgical candidates. The Technetium-99m labeled red blood cell scan has greater sensitivity at slow bleeding rates or for chronic bleeding sites and allows examination of the entire GI over an extended period of time. This increases the likelihood of a positive angiography from 22% to 53% [15].

## Conclusions

In summary, this case highlights the importance of keeping a high index of suspicion for the possibility and identification of more DLs in the vicinity of the first lesion, in order to minimize the chances of rebleeding from the missed lesions. Our case also emphasizes utilizing novel therapies like Hemospray for the control of bleeding if conventional endoscopic therapies fail to achieve initial hemostasis due to coagulopathy or the use of antiplatelets before considering more invasive options like angiography and surgery.
